# Sepsis and Its Impact on Outcomes in Elderly Patients Admitted to a Malaysian Intensive Care Unit

**DOI:** 10.21315/mjms2022.29.3.14

**Published:** 2022-06-28

**Authors:** Wan Fadzlina Wan Muhd Shukeri, Mohd Basri Mat Nor, Azrina Md Ralib

**Affiliations:** 1 Department of Anaesthesiology and Intensive Care, School of Medical Sciences, Universiti Sains Malaysia, Kelantan, Malaysia; 2 Department of Anaesthesiology and Critical Care, Kuliyyah of Medicine, International Islamic Universiti Malaysia, Pahang, Malaysia

**Keywords:** elderly, sepsis, septic shock, mortality, intensive care unit

## Abstract

Sepsis is an important cause of morbidity and mortality in elderly patients, but there is a scarcity of data on sepsis in this specific cohort. We performed this study to review the impact of sepsis on outcomes in elderly patients admitted to our local intensive care unit (ICU). This was a secondary analysis of prospectively collected data of 159 consecutive adult patients with sepsis admitted to an ICU of a tertiary hospital in Malaysia over a three-year period. Of the 159 patients analysed, elderly patients constituted 18.9% of the cohort. Fifty percent of the older patients died within 30 days, compared to 24% of younger patients (*P* = 0.005). On multivariate analysis, old age was found to be independently predictive of 30-day mortality with an adjusted odds ratio (OR) of 2.5 (95% confidence interval [CI]: 1.05, 6.01) compared to younger patients (*P* = 0.021). In a Kaplan-Meier analysis, survival probability was significantly lower in patients of an older age compared to younger patients (*P* = 0.015). In conclusion, mortality from sepsis is considerably higher in elderly patients, with age as an independent risk factor for mortality.

## Introduction

Sepsis, a syndrome of dysregulated host response to infection, is an important cause of morbidity and mortality in the older population (1). Unfortunately, studies on the diagnosis, management and prognostication of sepsis usually exclude the elderly cohort. In Malaysia, the burden of sepsis remains high, being the top reason for intensive care unit (ICU) admission nationwide, with an associated in-hospital mortality rate in excess of 50% (2). Despite the magnitude of such problems, there is a dearth of available local data on sepsis, particularly in the elderly cohort.

The aims of this study were (1) to describe the clinical characteristics, (2) to review the association of sepsis at ICU admission with mortality and other outcomes and (3) to assess the independent value of old age for mortality in critically ill elderly patients diagnosed with sepsis. There is no clear definition of elderly patients admitted to an ICU. In this manuscript and following the World Health Organization (WHO), the word ‘elderly’ was considered for an age frame of 65 years old or more.

## Methods

### Study Design and Participants

This was a secondary analysis of prospectively collected data performed in the ICU of a major tertiary hospital in Malaysia over a 3-year period. The aim of the original study was to assess the prognostic performance of a multi-marker approach in critically ill patients with sepsis and has been previously reported (3). A total of 159 patients who fulfilled the Sepsis-3 criteria were recruited. The protocol used in that study was approved by the local medical research and ethics committee and included consent for secondary analysis of the collected data.

### Data Collection

For the 159 patients, relevant baseline data were retrieved. These included the following: i) demographic data, i.e. age, sex and body mass index; ii) clinical data, i.e. admission category, the severity of illness measured as a baseline Simplified Acute Physiological Score II (SAPS II), a Sequential Organ Failure Assessment score, the presence of septic shock, the Charlson Comorbidity Index, primary sites of infection, the need for organ support and the proportion of patients who had limitations of life-sustaining therapy; iii) laboratory data, i.e. the presence of bacteraemia and baseline inflammatory biomarkers, including C-reactive protein, interleukin-6, procalcitonin and white blood cell count; iv) primary outcome data, i.e. 30-day mortality; and v) secondary outcome data, i.e. the duration of mechanical ventilation, the length of ICU stay and the length of hospital stay. We also calculated the modified SAPS II and Charlson Comorbidity Index by subtracting the point for age, to remove the impact of age on the severity of illness and comorbidities, respectively. For descriptive purposes, the proportions of ICU and in-hospital mortality were also described.

### Statistical Analysis

Continuous data are presented as mean with standard deviation (SD), while categorical data are presented as frequency (percentage). Patients were classified as elderly (aged 65 years old and above) and young (aged less than 65 years old). A comparison of continuous data was performed using an independent *t*-test, while a comparison of categorical data was performed using a chi-squared test. The independent value of old age for mortality was determined using binary logistic regression analysis, with 30-day mortality as the dependant variable and baseline characteristics with *P*-values less than 0.15 as covariates. The independent value of old age was expressed as an odds ratio (OR) with a 95% confidence interval (CI). The survival probability between the elderly and young groups was compared using Kaplan-Meier survival curves. *P*-values of less than 0.05 were considered statistically significant. Statistical analysis was performed using IBM SPSS version 24.0.

## Results

### Baseline Demographic, Clinical and Laboratory Characteristics

During the 3-year study period, the number of ICU admissions at our centre was 3,297, of which 276 (8.4%) patients were adults who were admitted with suspected sepsis. Among these 276 patients, 164 (59.4%) were recruited in the original study. For this analysis, 159 patients who fulfilled the revised Sepsis-3 criteria were studied.

The baseline characteristics of these 159 patients with sepsis are presented in Table 1. The elderly populations constituted 18.9% (*n* = 30) of the study population, of which one patient belonged in the very elderly group (aged more than 80 years old). The mean age was 71 (SD = 5) years old in the elderly group compared to 48 (SD = 16) years old in the young group (*P* < 0.0001). The elderly group was found to have a higher SAPS II of 51 (SD = 17) compared to 43 (SD = 16) in the young group (*P* = 0.021). However, after removing the point for age, there was no statistically significant difference between the groups in the modified SAPS II. The Charlson Comorbidity Index was also higher in the elderly group at 3.8 (SD = 1.6) compared to the young group at 1.3 (SD = 2.1) (*P* < 0.0001), but there was no statistically significant difference in the modified score, that is after removing the point for age. The most common site of infection in the elderly group was the lungs, similar to that of the young group. The two groups were also similar including in terms of the severity of organ failure, the severity of sepsis, the need for organ support and the level of baseline inflammatory biomarkers. Compared to the young group, the elderly group had more limitations of life-sustaining therapy (33.3% versus 4.7%, *P* < 0.0001), ICU mortality (26.7% versus 11.6%, *P* < 0.0001) and in-hospital mortality (46.7% versus 17.1%, *P* < 0.0001).

### Association of Older Age and Primary Outcome

**Figure 1:**
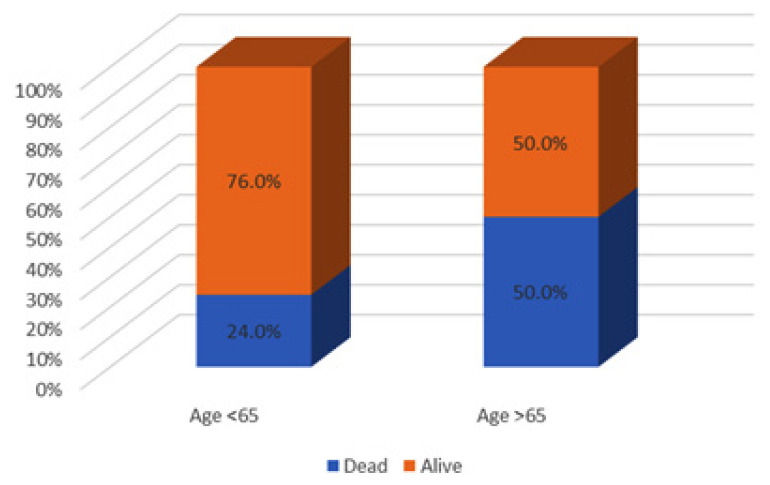
Association of older age with 30-day mortality in sepsis patients admitted to ICU

**Figure 2:**
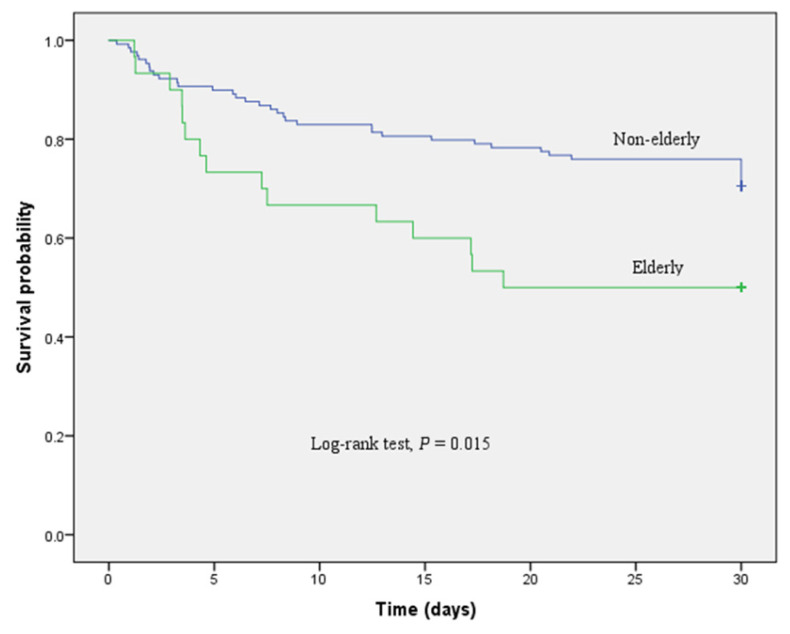
Kaplan-Meier plot showing survival probability in erderly versus non-erderly in the entire sepsis cohort

The primary outcome of 30-day mortality was reached in 46 out of 159 (28.9%) patients in this study. Of particular note, older age was associated with higher 30-day mortality, ranging from 31 out of 129 (24%) patients aged < 65 years old compared to 15 out of 30 (50%) patients aged ≥ 65 years old (*P* = 0.005) (Figure 1). After adjusting for sex and the severity of sepsis (septic shock), old age remains an independent predictor of mortality in sepsis with an adjusted OR of 2.51 (95% CI: 1.05, 6.01; *P* = 0.039) (Table 2). In addition to age, higher sepsis severity (septic shock) was also found to be independently predictive of 30-day mortality with an adjusted OR of 3.87 (95% CI: 1.76, 8.52; *P* = 0.001). In the Kaplan-Meier analysis, survival probability was significantly lower in patients of older age compared to younger patients (log rank test, *P* = 0.015) (Figure 2).

### Association of Older Age and Secondary Outcome

In this study, older age was not significantly associated with the secondary outcome of duration of mechanical ventilation, length of ICU stay or length of hospital stay.

## Discussion

This secondary analysis was performed with the primary intention of studying the impact of sepsis on mortality in elderly patients admitted to our local ICU. Several studies have shown that elderly patients admitted to the ICU have higher mortality rates as compared to their younger counterparts (4–5). Similarly, in our study, we found that patients aged 65 years old or older had a significantly higher 30-day mortality compared to young patients (50% versus 24%, *P* = 0.005) with sepsis.

Current evidence as to whether old age is associated with mortality in sepsis remains conflicted. In line with some studies by others (6–7), we found in our study that age is an independent risk factor for death, with an OR of dying of 2.5 as compared to young patients, after adjusting for potential confounders, including the severity of sepsis. This increase in mortality in the older population was also independent of comorbidities and the severity of illness at admission, as there was no significant difference in the modified Charlson Comorbidity Index and modified SAPS II between the old and young groups. In contrast, other studies have found no association between age and death (8–9), including a recent post-hoc analysis of the VIP1 multinational cohort in Europe whereby sepsis at admission was not independently associated with 30-day mortality in their very elderly ICU cohort (10). Various factors could have led to a different result being obtained in our local setting, such as variations in patient characteristics, implicated pathogens and their resistance pattern as well as differences in diagnostic and treatment modalities being practised in different settings.

The strength of our study is that it is focused on the septic subgroup (sepsis and septic shock) and to our knowledge, this is the first available evidence in the current literature which provides local data about outcomes for elderly patients with sepsis. The burden of sepsis is highest in low- and middle-income countries (11) but contemporary scientific evidence and guidelines on sepsis management almost exclusively originate in high-income countries. Most of the interventional sepsis trials invariably exclude elderly patients who are at a higher risk of death. Conversely, these are the individuals most likely to develop sepsis and thus should be the targets of such controlled investigations.

However, we must acknowledge that this study has a few pertinent limitations which have hindered us from performing a more comprehensive analysis in this field. First, a relatively small number of 159 sepsis patients were analysed, of which the elderly constituted only 18.9% of the study cohort. This proportion is far lower than the currently available global data, although these are almost exclusively reported in high-income countries. Second, the result in our study is from a single-centred data set, and as such, the question as to whether the study is generalisable to external populations is unknown. Third, as this was a secondary analysis, some important variables which may have influenced the outcome were not available for analysis. Further epidemiological studies are therefore warranted to determine the true incidence, risk factors and outcomes for the elderly patients being admitted with sepsis to our local ICU.

## Conclusion

This secondary analysis study shows that patients with sepsis aged 65 years old or over in our ICU had higher mortality rates compared to younger patients. Old age was found to be an independent risk factor for mortality in critically ill patients with sepsis, although this finding needs to be confirmed in a further prospective cohort study, which is currently ongoing at our centre.

## Figures and Tables

**Table 1:** Baseline demographics, clinical characteristics and outcome of the entire subjects

Variables	Young (*n* = 129)		Elderly (*n* = 30)		*P-*value
		
Demographic	Mean (SD)	*n* (%)	Mean (SD)	*n* (%)	
Age (years old)	48 (16)		71 (5)		< 0.0001
Sex (male)		85 (65.9)		24 (80.0)	0.134
BMI (kg/m^2^)	26.4 (7.2)		25.2 (3.8)		0.374
Clinical					
Admission category					0.972
Medical		95 (73.6)		22 (73.3)	
Surgical		34 (26.4)		8 (26.7)	
Severity of illness					
SAPS II	43 (16)		51 (17)		0.021
Modified SAPS II	39 (15)		37 (17)		0.564
SOFA	9 (4)		9 (5)		0.666
Septic shock		27 (20.9)		10 (33.3)	0.148
Comorbidities					
Charlson Comorbidity Index	1.3 (2.1)		3.8 (1.6)		< 0.0001
Modified Charlson Comorbidity	0.9 (1.9)		1.3 (1.6)		0.331
Index					
Primary sites of infection					
Lungs		72 (55.8)		18 (60.0)	0.677
Abdomen		12 (9.3)		3 (10.0)	0.906
Soft tissue		11 (8.5)		2 (6.7)	0.738
Urinary tract		6 (4.7)		3 (10.0)	0.253
Nervous system		8 (6.2)		-	0.162
Organ support					
Inotropic/vasopressor		27 (20.9)		10 (33.3)	0.148
Mechanical ventilation		122 (94.6)		29 (96.7)	0.637
RRT		41 (31.8)		11 (36.7)	0.608
Limitations of life-sustaining therapy		6 (4.7)		10 (33.3)	< 0.001
Laboratory					
Bacteraemia		21 (16.3)		6 (20.0)	0.625
CRP	73.8 (81.1)		102.8 (48.8)		0.479
IL-6	397.8 (355.8)		409.6 (379.9)		0.871
PCT	61.4 (173.2)		48.9 (107.4)		0.706
WBC	18.1 (10.6)		15.5 (7.7)		0.219
ICU-mortality		15 (11.6)		8 (26.7)	0.035
In-hospital mortality		22 (17.1)		14 (46.7)	< 0.001
30-day mortality		31 (24.0)		15 (50.0)	< 0.001

Notes: Data are expressed as mean (SD) or frequencies (%); The results of the comparison between the two groups were analysed by the independent *t*-test for continuous variables or the chi-squared test for categorical variables; BMI = body mass index; CRP = C-reactive protein; IL-6 = interleukin-6; PCT = procalcitonin; RRT = renal replacement therapy; SAPS II = Simplified Acute Physiological Score II; SOFA = Sequential Organ Failure Assessment; WBC = white blood cells count

**Table 2:** Independent value of old age for 30-day mortality after adjusting for sex, comorbidities and severity of sepsis

	Odd ratio	95% Confidence interval	*P*-value
Old age	2.51	1.05, 6.01	0.039
Male sex	1.65	0.71, 3.84	0.248
Septic shock	3.87	1.76, 8.52	0.001
